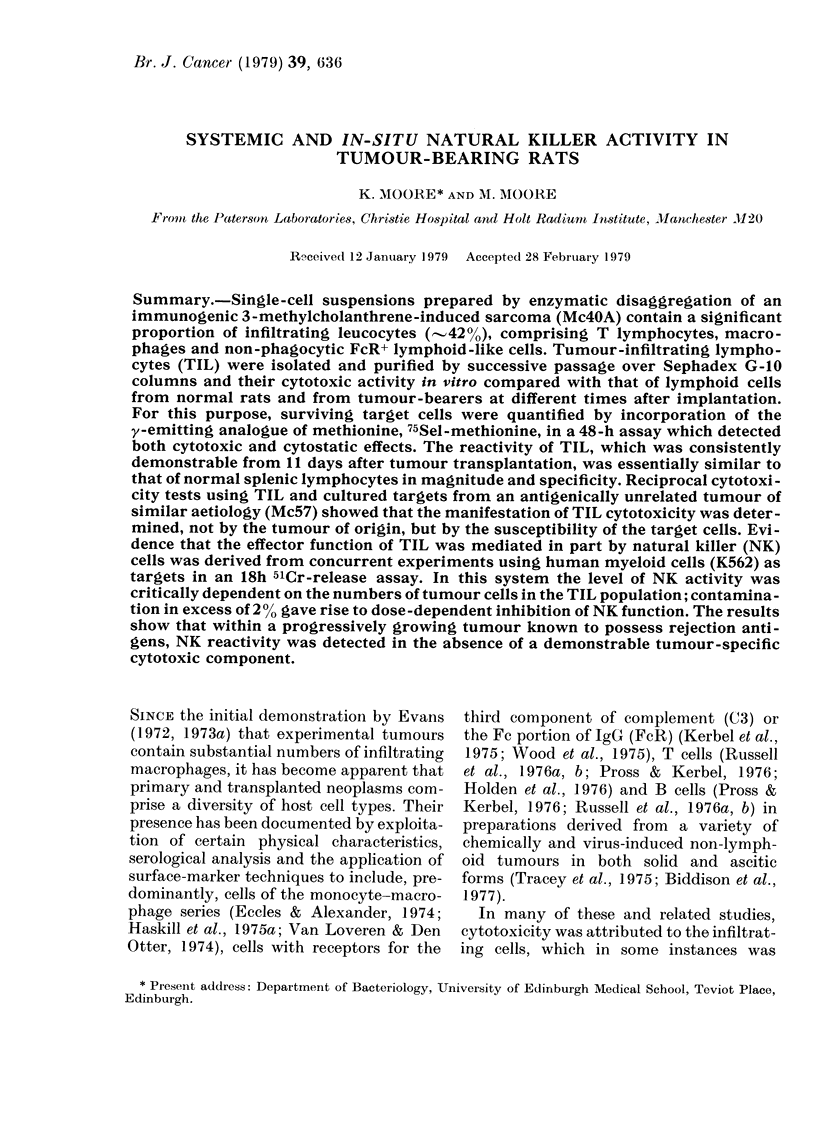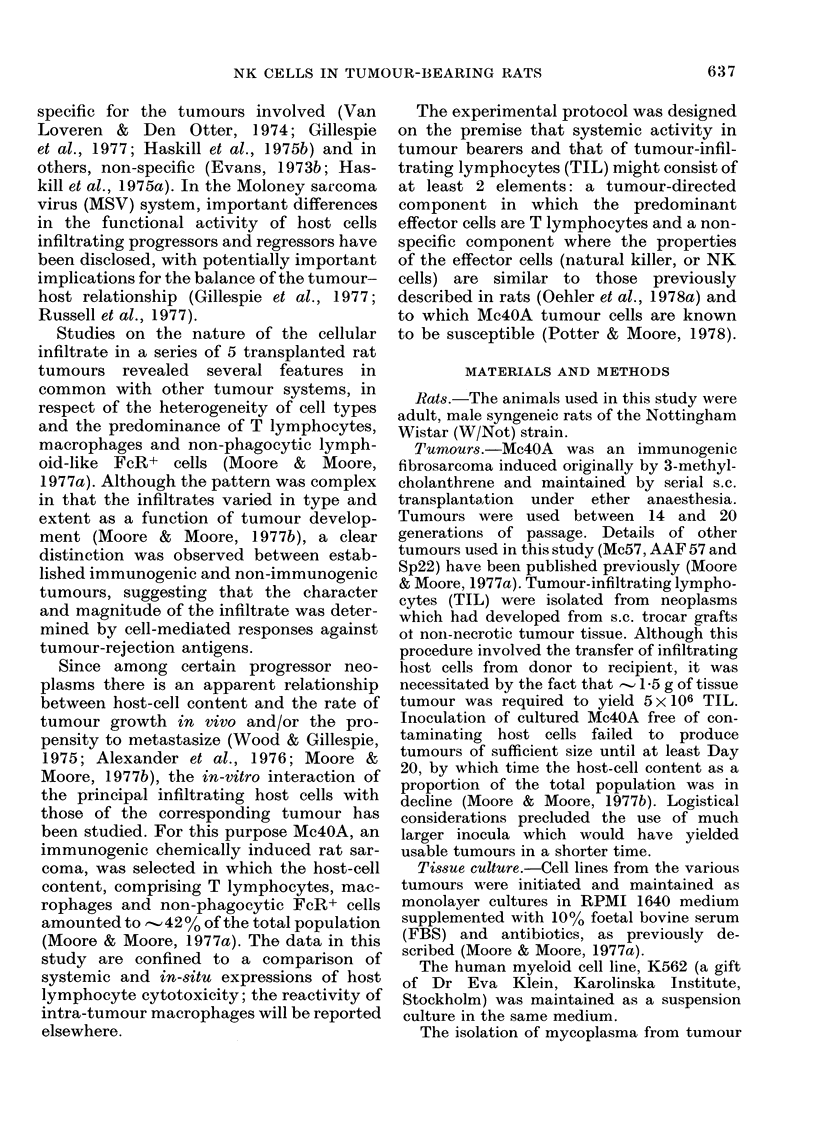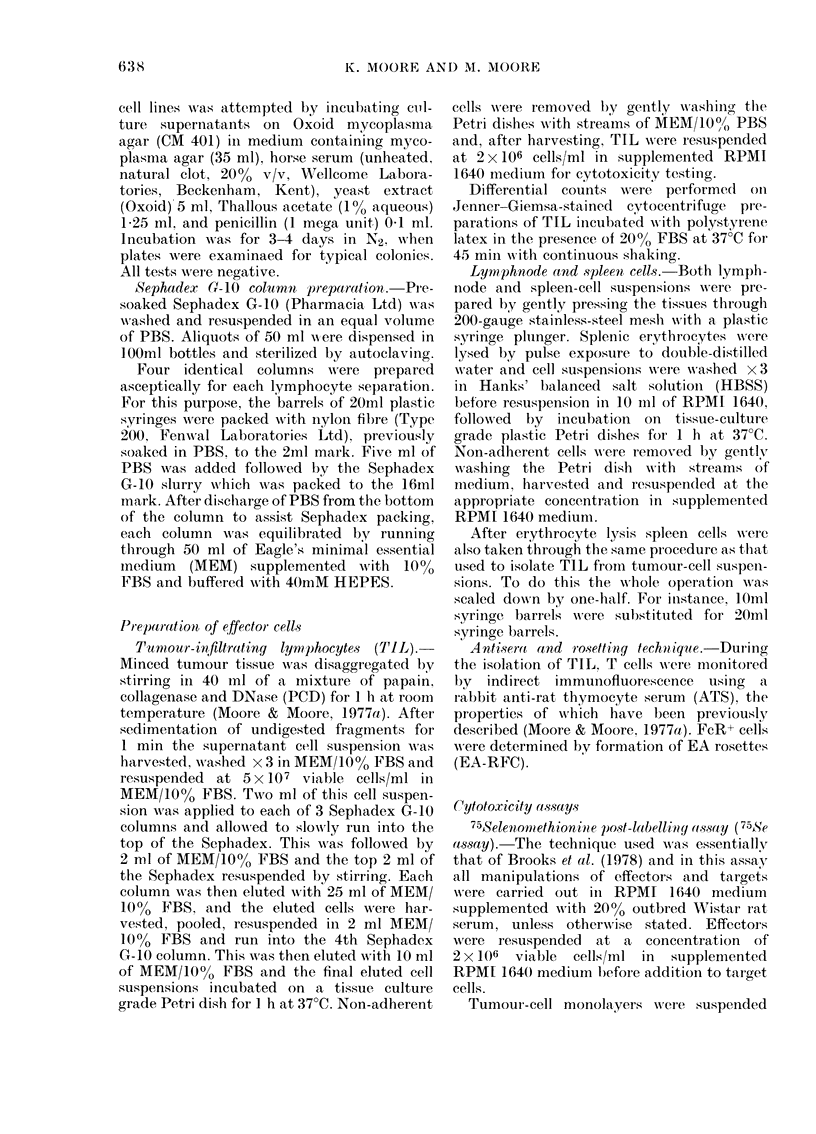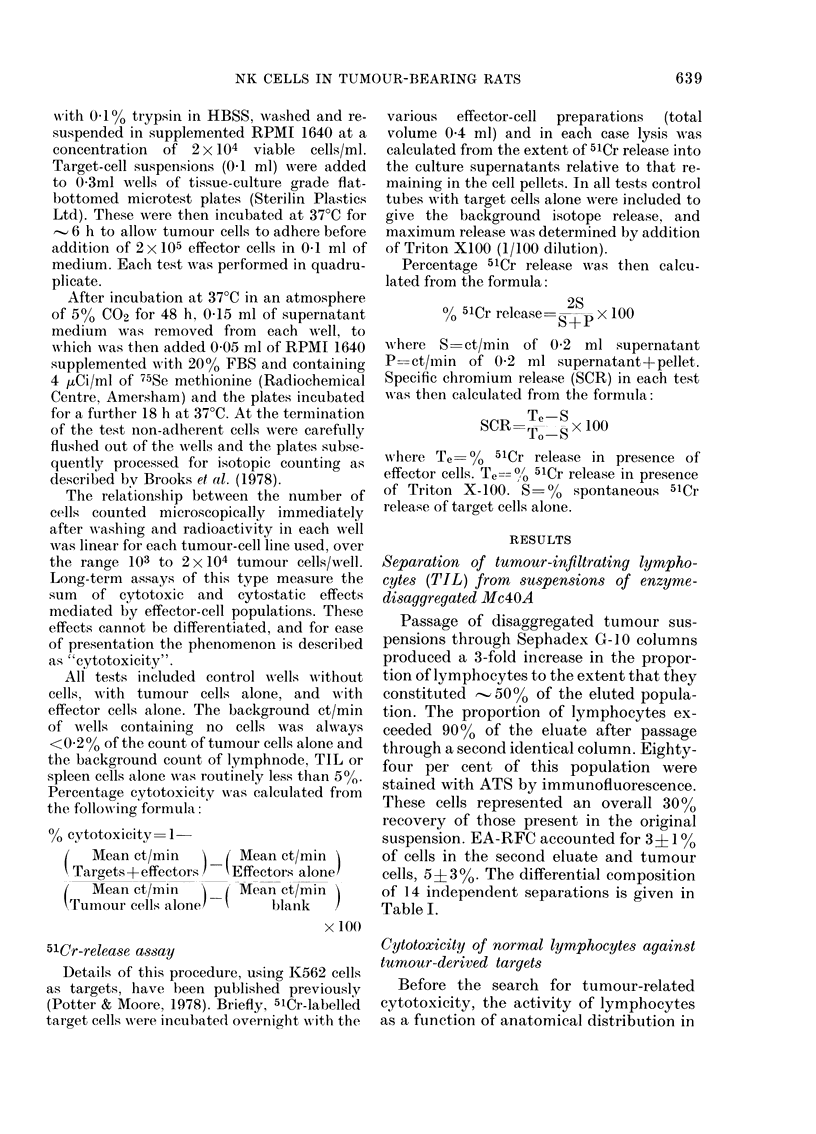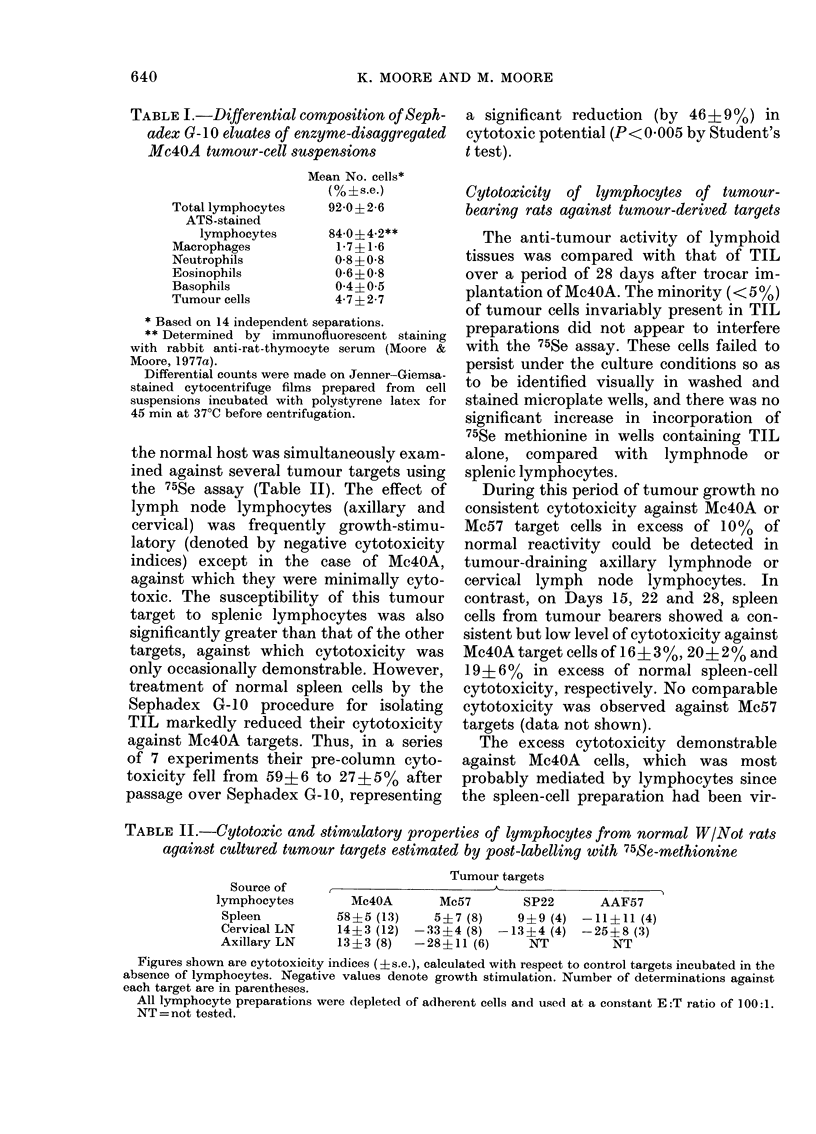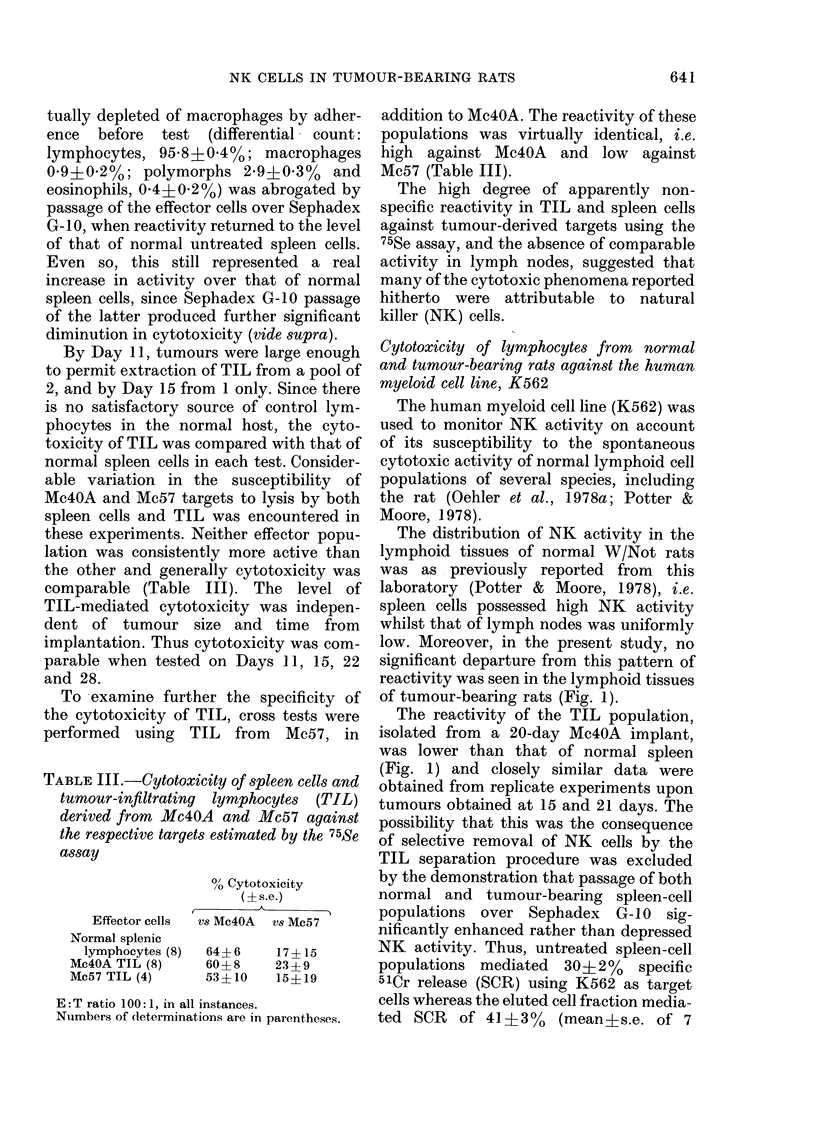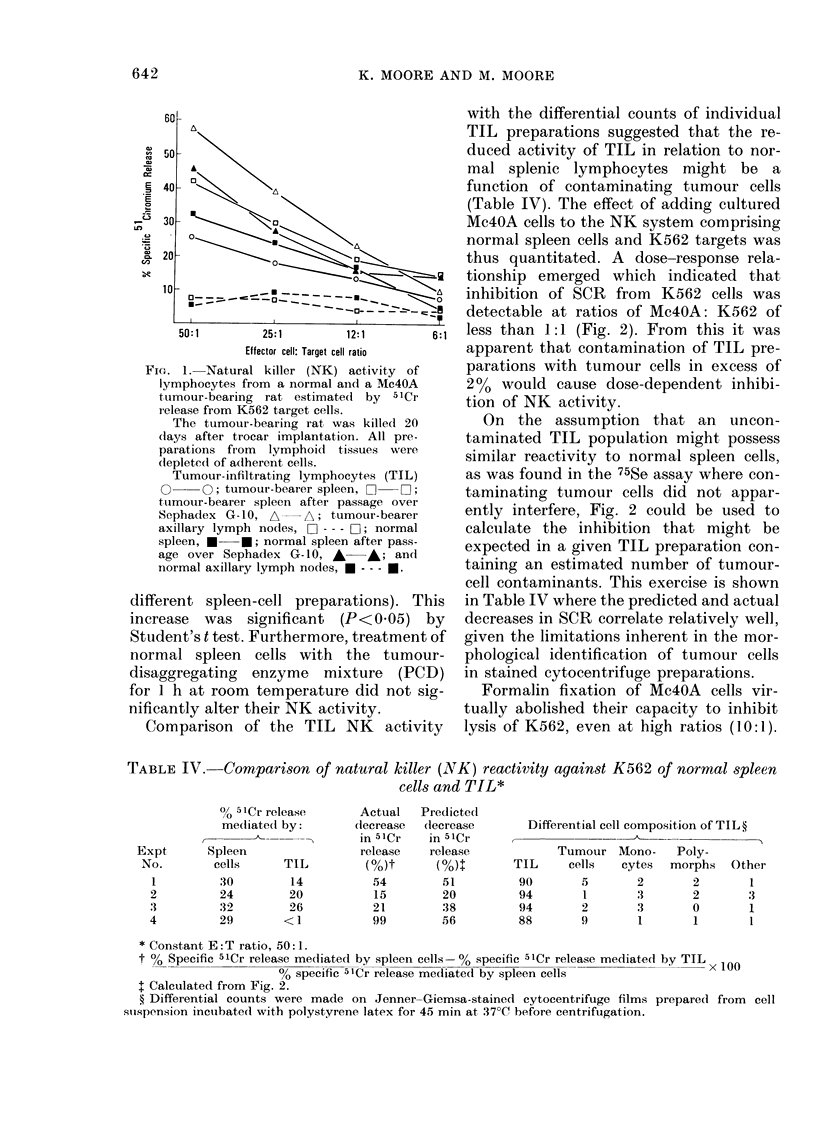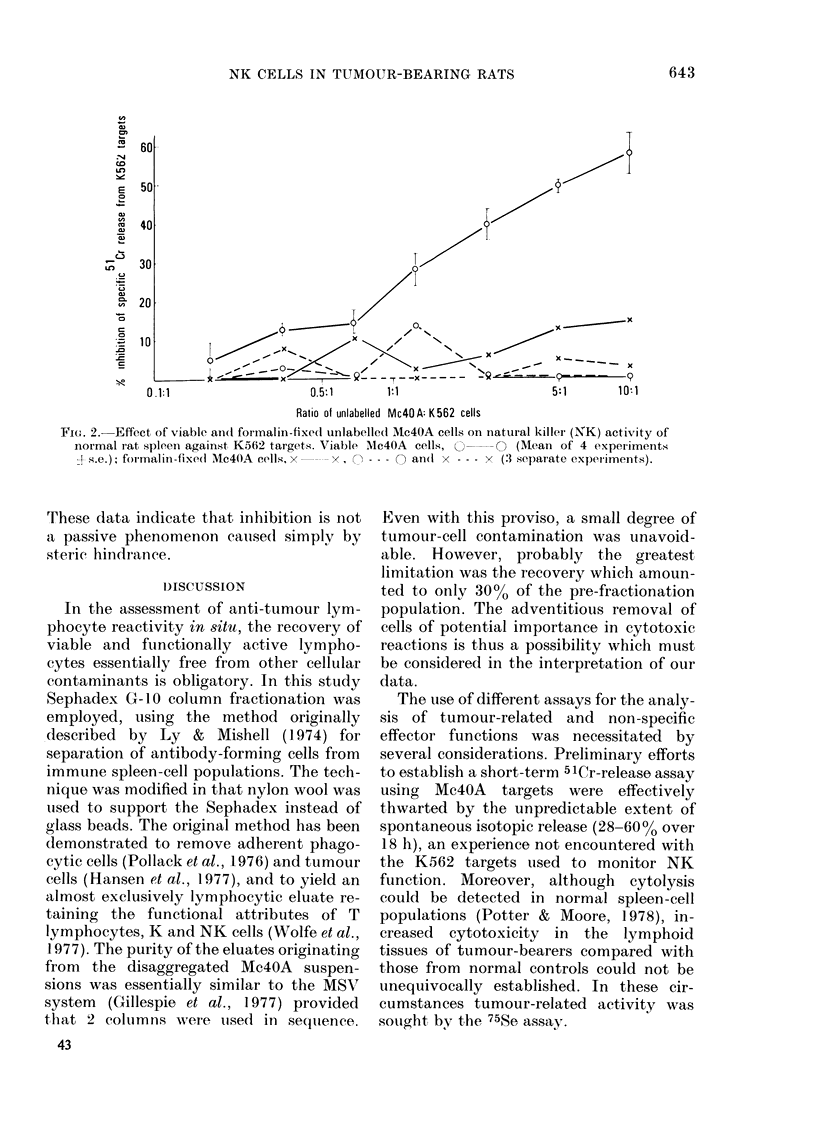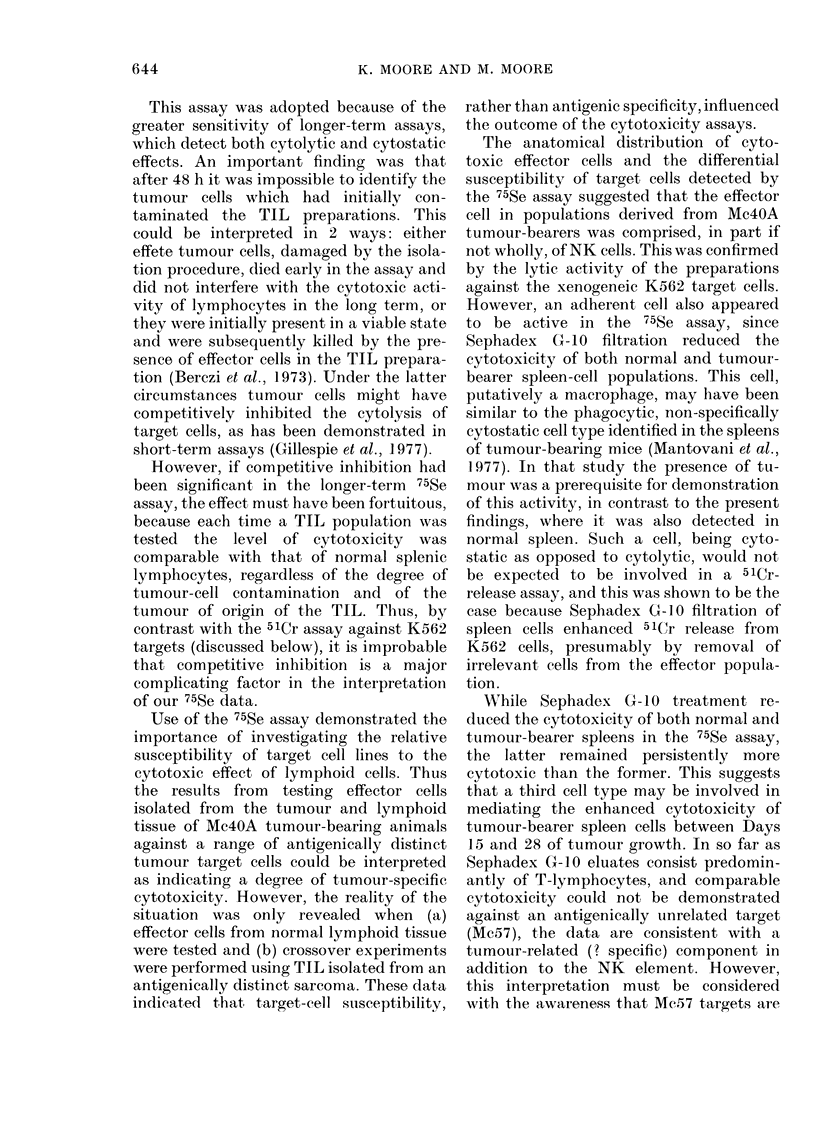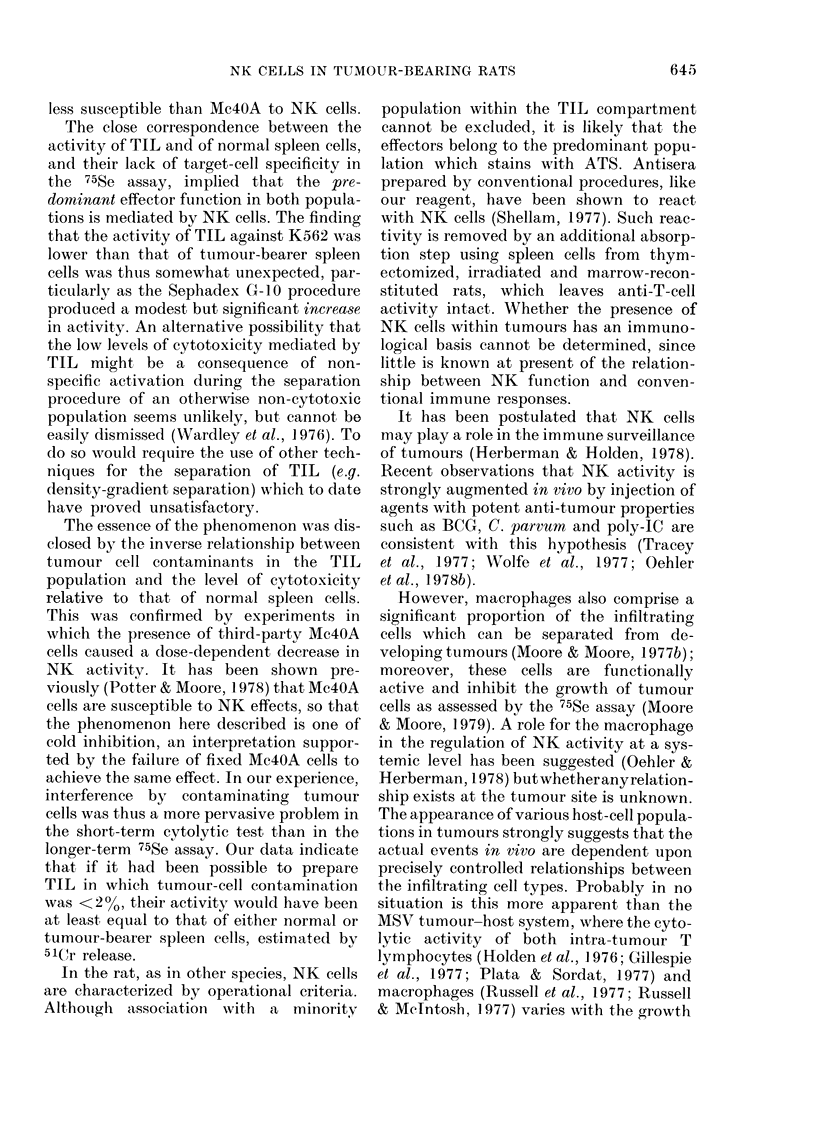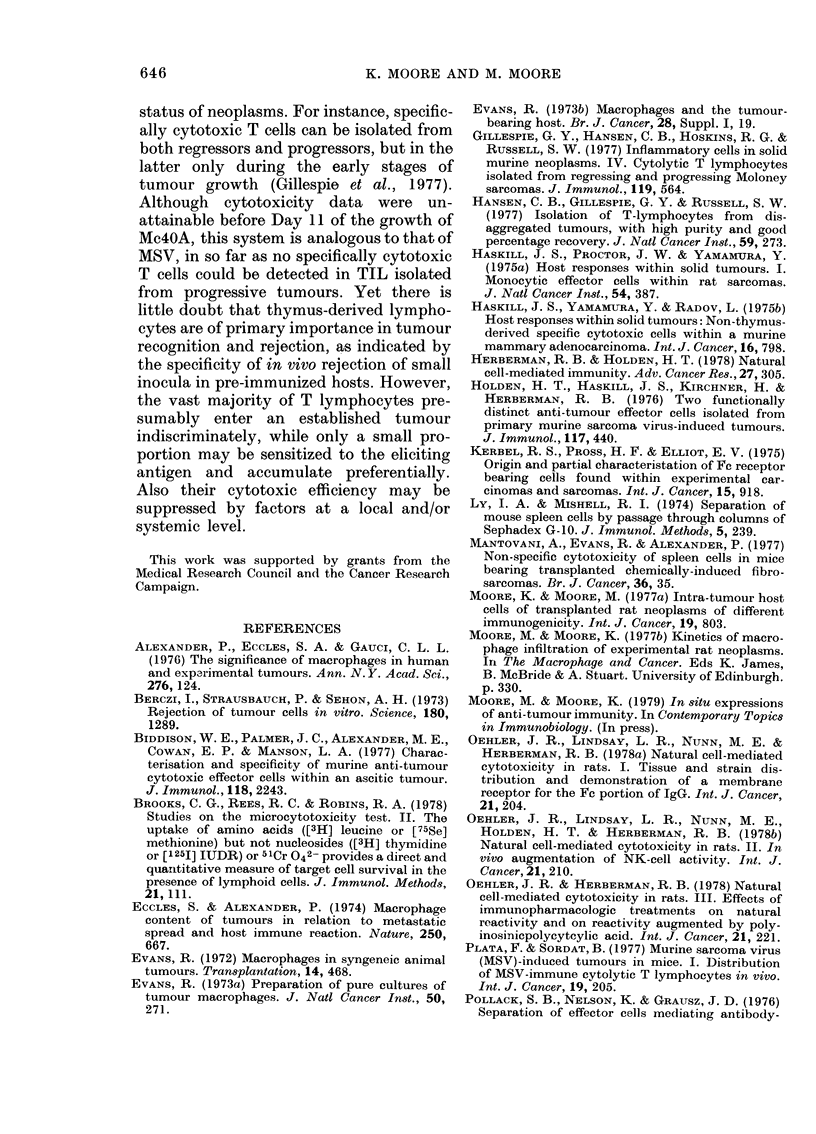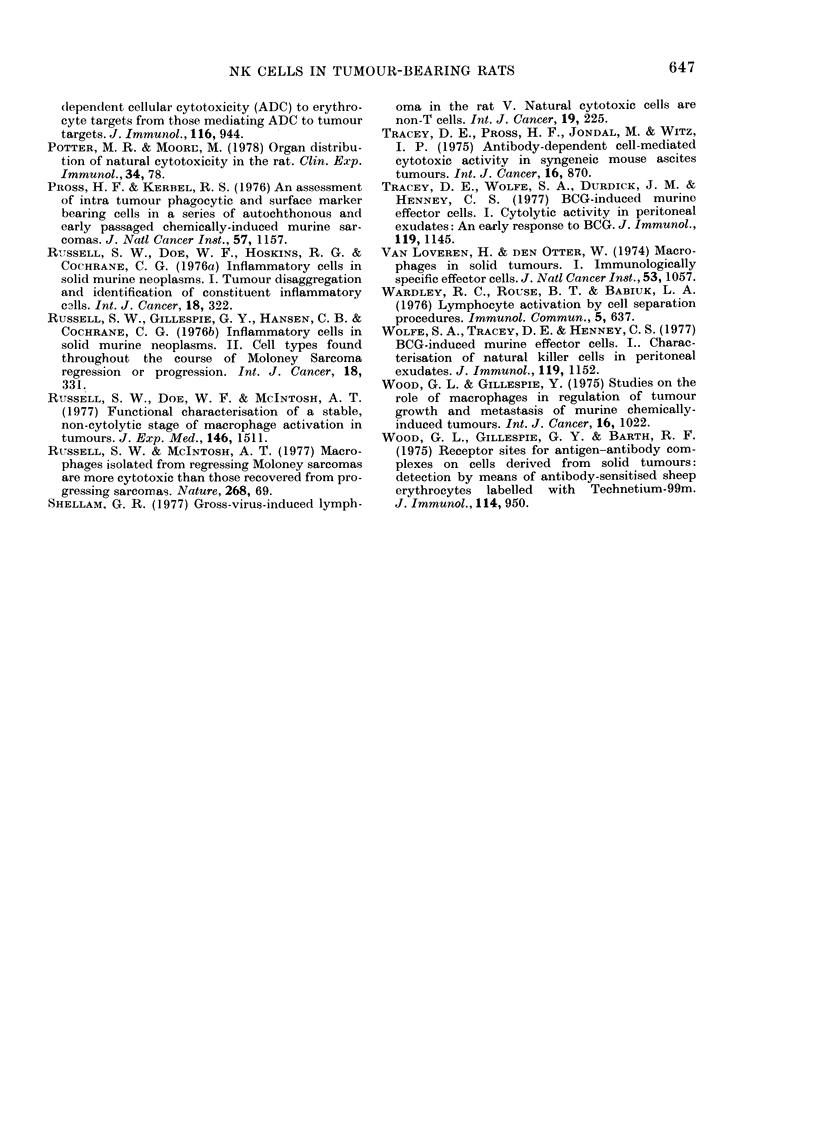# Systemic and in-situ natural killer activity in tumour-bearing rats.

**DOI:** 10.1038/bjc.1979.115

**Published:** 1979-06

**Authors:** K. Moore, M. Moore

## Abstract

Single-cell suspensions prepared by enzymatic disaggregation of an immunogenic 3-methylcholanthrene-induced sarcoma (Mc40A) contain a significant proportion of infiltrating leucocytes (approximately 42%), comprising T lymphocytes, macrophages and non-phagocytic FcR+ lymphoid-like cells. Tumour-infiltrating lymphocytes (TIL) were isolated and purified by successive passage over Sephadex G-10 columns and their cytotoxic activity in vitro compared with that of lymphoid cells from normal rats and from tumour-bearers at different times after implantation. For this purpose, surviving target cells were quantified by incorporation of the gamma-emitting analogue of methionine, 75Sel-methionine, in a 48-h assay which detected both cytotoxic and cytostatic effects. The reactivity of TIL, which was consistently demonstrable from 11 days after tumour transplantation, was essentially similar to that of normal splenic lymphocytes in magnitude and specificity. Reciprocal cytotoxicity tests using TIL and cultured targets from an antigenically unrelated tumour of similar aetiology (Mc57) showed that the manifestation of TIL cytotoxicity was determined, not by the tumour of origin, but by the susceptibility of the target cells. Evidence that the effector function of TIL was mediated in part by natural killer (NK) cells was derived from concurrent experiments using human myeloid cells (K562) as targets in an 18h 51Cr-release assay. In this system the level of NK activity was critically dependent on the numbers of tumour cells in the TIL population; contamination in excess of 2% gave rise to dose-dependent inhibition of NK function. The results show that within a progressively growing tumour known to possess rejection antigens, NK reactivity was detected in the absence of a demonstrable tumour-specific cytotoxic component.


*Br. J. Cancer* (1979) **39**, 636

### SYSTEMIC AND *IN-SITU* NATURAL KILLER ACTIVITY IN TUMOUR-BEARING RATS

K. MOORE* AND M. MOORE

*From the Paterson Laboratories, Christie Hospital and Holt Radium Institute, Manchester M20*

Received 12 January 1979 Accepted 28 February 1979

**Summary.—Single-cell suspensions prepared by enzymatic disaggregation of an immunogenic 3-methylcholanthrene-induced sarcoma (Mc40A) contain a significant proportion of infiltrating leucocytes (~42%), comprising T lymphocytes, macrophages and non-phagocytic $FcR^+$ lymphoid-like cells. Tumour-infiltrating lymphocytes (TIL) were isolated and purified by successive passage over Sephadex G-10 columns and their cytotoxic activity *in vitro* compared with that of lymphoid cells from normal rats and from tumour-bearers at different times after implantation. For this purpose, surviving target cells were quantified by incorporation of the γ-emitting analogue of methionine, $^{75}$Sel-methionine, in a 48-h assay which detected both cytotoxic and cytostatic effects. The reactivity of TIL, which was consistently demonstrable from 11 days after tumour transplantation, was essentially similar to that of normal splenic lymphocytes in magnitude and specificity. Reciprocal cytotoxicity tests using TIL and cultured targets from an antigenically unrelated tumour of similar aetiology (Mc57) showed that the manifestation of TIL cytotoxicity was determined, not by the tumour of origin, but by the susceptibility of the target cells. Evidence that the effector function of TIL was mediated in part by natural killer (NK) cells was derived from concurrent experiments using human myeloid cells (K562) as targets in an 18h $^{51}$Cr-release assay. In this system the level of NK activity was critically dependent on the numbers of tumour cells in the TIL population; contamination in excess of 2% gave rise to dose-dependent inhibition of NK function. The results show that within a progressively growing tumour known to possess rejection antigens, NK reactivity was detected in the absence of a demonstrable tumour-specific cytotoxic component.**

SINCE the initial demonstration by Evans (1972, 1973*a*) that experimental tumours contain substantial numbers of infiltrating macrophages, it has become apparent that primary and transplanted neoplasms comprise a diversity of host cell types. Their presence has been documented by exploitation of certain physical characteristics, serological analysis and the application of surface-marker techniques to include, predominantly, cells of the monocyte–macrophage series (Eccles & Alexander, 1974; Haskill *et al.*, 1975*a*; Van Loveren & Den Otter, 1974), cells with receptors for the third component of complement (C3) or the Fc portion of IgG (FcR) (Kerbel *et al.*, 1975; Wood *et al.*, 1975), T cells (Russell *et al.*, 1976*a*, *b*; Pross & Kerbel, 1976; Holden *et al.*, 1976) and B cells (Pross & Kerbel, 1976; Russell *et al.*, 1976*a*, *b*) in preparations derived from a variety of chemically and virus-induced non-lymphoid tumours in both solid and ascitic forms (Tracey *et al.*, 1975; Biddison *et al.*, 1977).

In many of these and related studies, cytotoxicity was attributed to the infiltrating cells, which in some instances was

* Present address: Department of Bacteriology, University of Edinburgh Medical School, Teviot Place, Edinburgh.

specific for the tumours involved (Van Loveren & Den Otter, 1974; Gillespie *et al.*, 1977; Haskill *et al.*, 1975*b*) and in others, non-specific (Evans, 1973*b*; Haskill *et al.*, 1975*a*). In the Moloney sarcoma virus (MSV) system, important differences in the functional activity of host cells infiltrating progressors and regressors have been disclosed, with potentially important implications for the balance of the tumour–host relationship (Gillespie *et al.*, 1977; Russell *et al.*, 1977).

Studies on the nature of the cellular infiltrate in a series of 5 transplanted rat tumours revealed several features in common with other tumour systems, in respect of the heterogeneity of cell types and the predominance of T lymphocytes, macrophages and non-phagocytic lymphoid-like $FcR^+$ cells (Moore & Moore, 1977*a*). Although the pattern was complex in that the infiltrates varied in type and extent as a function of tumour development (Moore & Moore, 1977*b*), a clear distinction was observed between established immunogenic and non-immunogenic tumours, suggesting that the character and magnitude of the infiltrate was determined by cell-mediated responses against tumour-rejection antigens.

Since among certain progressor neoplasms there is an apparent relationship between host-cell content and the rate of tumour growth *in vivo* and/or the propensity to metastasize (Wood & Gillespie, 1975; Alexander *et al.*, 1976; Moore & Moore, 1977*b*), the *in-vitro* interaction of the principal infiltrating host cells with those of the corresponding tumour has been studied. For this purpose Mc40A, an immunogenic chemically induced rat sarcoma, was selected in which the host-cell content, comprising T lymphocytes, macrophages and non-phagocytic $FcR^+$ cells amounted to $\sim 42\%$ of the total population (Moore & Moore, 1977*a*). The data in this study are confined to a comparison of systemic and *in-situ* expressions of host lymphocyte cytotoxicity; the reactivity of intra-tumour macrophages will be reported elsewhere.

The experimental protocol was designed on the premise that systemic activity in tumour bearers and that of tumour-infiltrating lymphocytes (TIL) might consist of at least 2 elements: a tumour-directed component in which the predominant effector cells are T lymphocytes and a non-specific component where the properties of the effector cells (natural killer, or NK cells) are similar to those previously described in rats (Oehler *et al.*, 1978*a*) and to which Mc40A tumour cells are known to be susceptible (Potter & Moore, 1978).

### MATERIALS AND METHODS

*Rats.*—The animals used in this study were adult, male syngeneic rats of the Nottingham Wistar (W/Not) strain.

*Tumours.*—Mc40A was an immunogenic fibrosarcoma induced originally by 3-methylcholanthrene and maintained by serial s.c. transplantation under ether anaesthesia. Tumours were used between 14 and 20 generations of passage. Details of other tumours used in this study (Mc57, AAF 57 and Sp22) have been published previously (Moore & Moore, 1977*a*). Tumour-infiltrating lymphocytes (TIL) were isolated from neoplasms which had developed from s.c. trocar grafts of non-necrotic tumour tissue. Although this procedure involved the transfer of infiltrating host cells from donor to recipient, it was necessitated by the fact that $\sim 1{\cdot}5$ g of tissue tumour was required to yield $5 \times 10^6$ TIL. Inoculation of cultured Mc40A free of contaminating host cells failed to produce tumours of sufficient size until at least Day 20, by which time the host-cell content as a proportion of the total population was in decline (Moore & Moore, 1977*b*). Logistical considerations precluded the use of much larger inocula which would have yielded usable tumours in a shorter time.

*Tissue culture.*—Cell lines from the various tumours were initiated and maintained as monolayer cultures in RPMI 1640 medium supplemented with 10% foetal bovine serum (FBS) and antibiotics, as previously described (Moore & Moore, 1977*a*).

The human myeloid cell line, K562 (a gift of Dr Eva Klein, Karolinska Institute, Stockholm) was maintained as a suspension culture in the same medium.

The isolation of mycoplasma from tumour cell lines was attempted by incubating culture supernatants on Oxoid mycoplasma agar (CM 401) in medium containing mycoplasma agar (35 ml), horse serum (unheated, natural clot, 20% v/v, Wellcome Laboratories, Beckenham, Kent), yeast extract (Oxoid) 5 ml, Thallous acetate (1% aqueous) 1·25 ml, and penicillin (1 mega unit) 0·1 ml. Incubation was for 3–4 days in $N_2$, when plates were examinaed for typical colonies. All tests were negative.

*Sephadex G-10 column preparation.*—Presoaked Sephadex G-10 (Pharmacia Ltd) was washed and resuspended in an equal volume of PBS. Aliquots of 50 ml were dispensed in 100ml bottles and sterilized by autoclaving.

Four identical columns were prepared asceptically for each lymphocyte separation. For this purpose, the barrels of 20ml plastic syringes were packed with nylon fibre (Type 200, Fenwal Laboratories Ltd), previously soaked in PBS, to the 2ml mark. Five ml of PBS was added followed by the Sephadex G-10 slurry which was packed to the 16ml mark. After discharge of PBS from the bottom of the column to assist Sephadex packing, each column was equilibrated by running through 50 ml of Eagle's minimal essential medium (MEM) supplemented with 10% FBS and buffered with 40mM HEPES.

### *Preparation of effector cells*

*Tumour-infiltrating lymphocytes (TIL).*—Minced tumour tissue was disaggregated by stirring in 40 ml of a mixture of papain, collagenase and DNase (PCD) for 1 h at room temperature (Moore & Moore, 1977*a*). After sedimentation of undigested fragments for 1 min the supernatant cell suspension was harvested, washed $\times 3$ in MEM/10% FBS and resuspended at $5 \times 10^7$ viable cells/ml in MEM/10% FBS. Two ml of this cell suspension was applied to each of 3 Sephadex G-10 columns and allowed to slowly run into the top of the Sephadex. This was followed by 2 ml of MEM/10% FBS and the top 2 ml of the Sephadex resuspended by stirring. Each column was then eluted with 25 ml of MEM/10% FBS, and the eluted cells were harvested, pooled, resuspended in 2 ml MEM/10% FBS and run into the 4th Sephadex G-10 column. This was then eluted with 10 ml of MEM/10% FBS and the final eluted cell suspensions incubated on a tissue culture grade Petri dish for 1 h at 37°C. Non-adherent cells were removed by gently washing the Petri dishes with streams of MEM/10% PBS and, after harvesting, TIL were resuspended at $2 \times 10^6$ cells/ml in supplemented RPMI 1640 medium for cytotoxicity testing.

Differential counts were performed on Jenner–Giemsa-stained cytocentrifuge preparations of TIL incubated with polystyrene latex in the presence of 20% FBS at 37°C for 45 min with continuous shaking.

*Lymphnode and spleen cells.*—Both lymphnode and spleen-cell suspensions were prepared by gently pressing the tissues through 200-gauge stainless-steel mesh with a plastic syringe plunger. Splenic erythrocytes were lysed by pulse exposure to double-distilled water and cell suspensions were washed $\times 3$ in Hanks' balanced salt solution (HBSS) before resuspension in 10 ml of RPMI 1640, followed by incubation on tissue-culture grade plastic Petri dishes for 1 h at 37°C. Non-adherent cells were removed by gently washing the Petri dish with streams of medium, harvested and resuspended at the appropriate concentration in supplemented RPMI 1640 medium.

After erythrocyte lysis spleen cells were also taken through the same procedure as that used to isolate TIL from tumour-cell suspensions. To do this the whole operation was scaled down by one-half. For instance, 10ml syringe barrels were substituted for 20ml syringe barrels.

*Antisera and rosetting technique.*—During the isolation of TIL, T cells were monitored by indirect immunofluorescence using a rabbit anti-rat thymocyte serum (ATS), the properties of which have been previously described (Moore & Moore, 1977*a*). $FcR^+$ cells were determined by formation of EA rosettes (EA-RFC).

### *Cytotoxicity assays*

*$^{75}$Selenomethionine post-labelling assay ($^{75}$Se assay).*—The technique used was essentially that of Brooks *et al.* (1978) and in this assay all manipulations of effectors and targets were carried out in RPMI 1640 medium supplemented with 20% outbred Wistar rat serum, unless otherwise stated. Effectors were resuspended at a concentration of $2 \times 10^6$ viable cells/ml in supplemented RPMI 1640 medium before addition to target cells.

Tumour-cell monolayers were suspended with 0·1% trypsin in HBSS, washed and resuspended in supplemented RPMI 1640 at a concentration of $2\times10^4$ viable cells/ml. Target-cell suspensions (0·1 ml) were added to 0·3ml wells of tissue-culture grade flat-bottomed microtest plates (Sterilin Plastics Ltd). These were then incubated at 37°C for ~6 h to allow tumour cells to adhere before addition of $2\times10^5$ effector cells in 0·1 ml of medium. Each test was performed in quadruplicate.

After incubation at 37°C in an atmosphere of 5% $CO_2$ for 48 h, 0·15 ml of supernatant medium was removed from each well, to which was then added 0·05 ml of RPMI 1640 supplemented with 20% FBS and containing 4 $\mu$Ci/ml of $^{75}$Se methionine (Radiochemical Centre, Amersham) and the plates incubated for a further 18 h at 37°C. At the termination of the test non-adherent cells were carefully flushed out of the wells and the plates subsequently processed for isotopic counting as described by Brooks *et al.* (1978).

The relationship between the number of cells counted microscopically immediately after washing and radioactivity in each well was linear for each tumour-cell line used, over the range $10^3$ to $2\times10^4$ tumour cells/well. Long-term assays of this type measure the sum of cytotoxic and cytostatic effects mediated by effector-cell populations. These effects cannot be differentiated, and for ease of presentation the phenomenon is described as "cytotoxicity".

All tests included control wells without cells, with tumour cells alone, and with effector cells alone. The background ct/min of wells containing no cells was always <0·2% of the count of tumour cells alone and the background count of lymphnode, TIL or spleen cells alone was routinely less than 5%. Percentage cytotoxicity was calculated from the following formula:

$$\%\ \text{cytotoxicity} = 1 - \frac{\begin{pmatrix}\text{Mean ct/min}\\ \text{Targets+effectors}\end{pmatrix} - \begin{pmatrix}\text{Mean ct/min}\\ \text{Effectors alone}\end{pmatrix}}{\begin{pmatrix}\text{Mean ct/min}\\ \text{Tumour cells alone}\end{pmatrix} - \begin{pmatrix}\text{Mean ct/min}\\ \text{blank}\end{pmatrix}} \times 100$$

### $^{51}$*Cr-release assay*

Details of this procedure, using K562 cells as targets, have been published previously (Potter & Moore, 1978). Briefly, $^{51}$Cr-labelled target cells were incubated overnight with the various effector-cell preparations (total volume 0·4 ml) and in each case lysis was calculated from the extent of $^{51}$Cr release into the culture supernatants relative to that remaining in the cell pellets. In all tests control tubes with target cells alone were included to give the background isotope release, and maximum release was determined by addition of Triton X100 (1/100 dilution).

Percentage $^{51}$Cr release was then calculated from the formula:

$$\%\ ^{51}\text{Cr release} = \frac{2\text{S}}{\text{S}+\text{P}} \times 100$$

where S=ct/min of 0·2 ml supernatant P=ct/min of 0·2 ml supernatant+pellet. Specific chromium release (SCR) in each test was then calculated from the formula:

$$\text{SCR} = \frac{\text{T}_\text{e}-\text{S}}{\text{T}_\text{o}-\text{S}} \times 100$$

where $T_e$=% $^{51}$Cr release in presence of effector cells. $T_e$=% $^{51}$Cr release in presence of Triton X-100. S=% spontaneous $^{51}$Cr release of target cells alone.

### RESULTS

### *Separation of tumour-infiltrating lymphocytes (TIL) from suspensions of enzyme-disaggregated Mc40A*

Passage of disaggregated tumour suspensions through Sephadex G-10 columns produced a 3-fold increase in the proportion of lymphocytes to the extent that they constituted ~50% of the eluted population. The proportion of lymphocytes exceeded 90% of the eluate after passage through a second identical column. Eighty-four per cent of this population were stained with ATS by immunofluorescence. These cells represented an overall 30% recovery of those present in the original suspension. EA-RFC accounted for 3±1% of cells in the second eluate and tumour cells, 5±3%. The differential composition of 14 independent separations is given in Table I.

### *Cytotoxicity of normal lymphocytes against tumour-derived targets*

Before the search for tumour-related cytotoxicity, the activity of lymphocytes as a function of anatomical distribution in

TABLE I.—*Differential composition of Sephadex G-10 eluates of enzyme-disaggregated Mc40A tumour-cell suspensions*

| | Mean No. cells* (% ± s.e.) |
|---|---|
| Total lymphocytes | 92·0 ± 2·6 |
| ATS-stained lymphocytes | 84·0 ± 4·2** |
| Macrophages | 1·7 ± 1·6 |
| Neutrophils | 0·8 ± 0·8 |
| Eosinophils | 0·6 ± 0·8 |
| Basophils | 0·4 ± 0·5 |
| Tumour cells | 4·7 ± 2·7 |

* Based on 14 independent separations.

** Determined by immunofluorescent staining with rabbit anti-rat-thymocyte serum (Moore & Moore, 1977*a*).

Differential counts were made on Jenner–Giemsa-stained cytocentrifuge films prepared from cell suspensions incubated with polystyrene latex for 45 min at 37°C before centrifugation.

the normal host was simultaneously examined against several tumour targets using the $^{75}$Se assay (Table II). The effect of lymph node lymphocytes (axillary and cervical) was frequently growth-stimulatory (denoted by negative cytotoxicity indices) except in the case of Mc40A, against which they were minimally cytotoxic. The susceptibility of this tumour target to splenic lymphocytes was also significantly greater than that of the other targets, against which cytotoxicity was only occasionally demonstrable. However, treatment of normal spleen cells by the Sephadex G-10 procedure for isolating TIL markedly reduced their cytotoxicity against Mc40A targets. Thus, in a series of 7 experiments their pre-column cytotoxicity fell from 59 ± 6 to 27 ± 5% after passage over Sephadex G-10, representing a significant reduction (by 46 ± 9%) in cytotoxic potential ($P < 0·005$ by Student's *t* test).

*Cytotoxicity of lymphocytes of tumour-bearing rats against tumour-derived targets*

The anti-tumour activity of lymphoid tissues was compared with that of TIL over a period of 28 days after trocar implantation of Mc40A. The minority (<5%) of tumour cells invariably present in TIL preparations did not appear to interfere with the $^{75}$Se assay. These cells failed to persist under the culture conditions so as to be identified visually in washed and stained microplate wells, and there was no significant increase in incorporation of $^{75}$Se methionine in wells containing TIL alone, compared with lymphnode or splenic lymphocytes.

During this period of tumour growth no consistent cytotoxicity against Mc40A or Mc57 target cells in excess of 10% of normal reactivity could be detected in tumour-draining axillary lymphnode or cervical lymph node lymphocytes. In contrast, on Days 15, 22 and 28, spleen cells from tumour bearers showed a consistent but low level of cytotoxicity against Mc40A target cells of 16 ± 3%, 20 ± 2% and 19 ± 6% in excess of normal spleen-cell cytotoxicity, respectively. No comparable cytotoxicity was observed against Mc57 targets (data not shown).

The excess cytotoxicity demonstrable against Mc40A cells, which was most probably mediated by lymphocytes since the spleen-cell preparation had been vir-

TABLE II.—*Cytotoxic and stimulatory properties of lymphocytes from normal W/Not rats against cultured tumour targets estimated by post-labelling with* $^{75}$*Se-methionine*

| Source of lymphocytes | Tumour targets | | | |
|---|---|---|---|---|
| | Mc40A | Mc57 | SP22 | AAF57 |
| Spleen | 58 ± 5 (13) | 5 ± 7 (8) | 9 ± 9 (4) | −11 ± 11 (4) |
| Cervical LN | 14 ± 3 (12) | −33 ± 4 (8) | −13 ± 4 (4) | −25 ± 8 (3) |
| Axillary LN | 13 ± 3 (8) | −28 ± 11 (6) | NT | NT |

Figures shown are cytotoxicity indices (± s.e.), calculated with respect to control targets incubated in the absence of lymphocytes. Negative values denote growth stimulation. Number of determinations against each target are in parentheses.

All lymphocyte preparations were depleted of adherent cells and used at a constant E:T ratio of 100:1. NT = not tested.

tually depleted of macrophages by adherence before test (differential count: lymphocytes, 95·8±0·4%; macrophages 0·9±0·2%; polymorphs 2·9±0·3% and eosinophils, 0·4±0·2%) was abrogated by passage of the effector cells over Sephadex G-10, when reactivity returned to the level of that of normal untreated spleen cells. Even so, this still represented a real increase in activity over that of normal spleen cells, since Sephadex G-10 passage of the latter produced further significant diminution in cytotoxicity (*vide supra*).

By Day 11, tumours were large enough to permit extraction of TIL from a pool of 2, and by Day 15 from 1 only. Since there is no satisfactory source of control lymphocytes in the normal host, the cytotoxicity of TIL was compared with that of normal spleen cells in each test. Considerable variation in the susceptibility of Mc40A and Mc57 targets to lysis by both spleen cells and TIL was encountered in these experiments. Neither effector population was consistently more active than the other and generally cytotoxicity was comparable (Table III). The level of TIL-mediated cytotoxicity was independent of tumour size and time from implantation. Thus cytotoxicity was comparable when tested on Days 11, 15, 22 and 28.

To examine further the specificity of the cytotoxicity of TIL, cross tests were performed using TIL from Mc57, in addition to Mc40A. The reactivity of these populations was virtually identical, *i.e.* high against Mc40A and low against Mc57 (Table III).

The high degree of apparently nonspecific reactivity in TIL and spleen cells against tumour-derived targets using the $^{75}$Se assay, and the absence of comparable activity in lymph nodes, suggested that many of the cytotoxic phenomena reported hitherto were attributable to natural killer (NK) cells.

TABLE III.—*Cytotoxicity of spleen cells and tumour-infiltrating lymphocytes (TIL) derived from Mc40A and Mc57 against the respective targets estimated by the $^{75}$Se assay*

| | % Cytotoxicity (±s.e.) | |
|---|---|---|
| Effector cells | *vs* Mc40A | *vs* Mc57 |
| Normal splenic lymphocytes (8) | 64±6 | 17±15 |
| Mc40A TIL (8) | 60±8 | 23±9 |
| Mc57 TIL (4) | 53±10 | 15±19 |

E:T ratio 100:1, in all instances.
Numbers of determinations are in parentheses.

*Cytotoxicity of lymphocytes from normal and tumour-bearing rats against the human myeloid cell line, K562*

The human myeloid cell line (K562) was used to monitor NK activity on account of its susceptibility to the spontaneous cytotoxic activity of normal lymphoid cell populations of several species, including the rat (Oehler *et al.*, 1978*a*; Potter & Moore, 1978).

The distribution of NK activity in the lymphoid tissues of normal W/Not rats was as previously reported from this laboratory (Potter & Moore, 1978), *i.e.* spleen cells possessed high NK activity whilst that of lymph nodes was uniformly low. Moreover, in the present study, no significant departure from this pattern of reactivity was seen in the lymphoid tissues of tumour-bearing rats (Fig. 1).

The reactivity of the TIL population, isolated from a 20-day Mc40A implant, was lower than that of normal spleen (Fig. 1) and closely similar data were obtained from replicate experiments upon tumours obtained at 15 and 21 days. The possibility that this was the consequence of selective removal of NK cells by the TIL separation procedure was excluded by the demonstration that passage of both normal and tumour-bearing spleen-cell populations over Sephadex G-10 significantly enhanced rather than depressed NK activity. Thus, untreated spleen-cell populations mediated 30±2% specific $^{51}$Cr release (SCR) using K562 as target cells whereas the eluted cell fraction mediated SCR of 41±3% (mean±s.e. of 7

FIG. 1.—Natural killer (NK) activity of lymphocytes from a normal and a Mc40A tumour-bearing rat estimated by $^{51}Cr$ release from K562 target cells.

The tumour-bearing rat was killed 20 days after trocar implantation. All preparations from lymphoid tissues were depleted of adherent cells.

Tumour-infiltrating lymphocytes (TIL) ○——○; tumour-bearer spleen, □——□; tumour-bearer spleen after passage over Sephadex G-10, △——△; tumour-bearer axillary lymph nodes, □ - - - □; normal spleen, ■——■; normal spleen after passage over Sephadex G-10, ▲——▲; and normal axillary lymph nodes, ■ - - - ■.

different spleen-cell preparations). This increase was significant ($P<0{\cdot}05$) by Student's $t$ test. Furthermore, treatment of normal spleen cells with the tumour-disaggregating enzyme mixture (PCD) for 1 h at room temperature did not significantly alter their NK activity.

Comparison of the TIL NK activity with the differential counts of individual TIL preparations suggested that the reduced activity of TIL in relation to normal splenic lymphocytes might be a function of contaminating tumour cells (Table IV). The effect of adding cultured Mc40A cells to the NK system comprising normal spleen cells and K562 targets was thus quantitated. A dose–response relationship emerged which indicated that inhibition of SCR from K562 cells was detectable at ratios of Mc40A: K562 of less than 1:1 (Fig. 2). From this it was apparent that contamination of TIL preparations with tumour cells in excess of 2% would cause dose-dependent inhibition of NK activity.

On the assumption that an uncontaminated TIL population might possess similar reactivity to normal spleen cells, as was found in the $^{75}Se$ assay where contaminating tumour cells did not apparently interfere, Fig. 2 could be used to calculate the inhibition that might be expected in a given TIL preparation containing an estimated number of tumour-cell contaminants. This exercise is shown in Table IV where the predicted and actual decreases in SCR correlate relatively well, given the limitations inherent in the morphological identification of tumour cells in stained cytocentrifuge preparations.

Formalin fixation of Mc40A cells virtually abolished their capacity to inhibit lysis of K562, even at high ratios (10:1).

TABLE IV.—*Comparison of natural killer (NK) reactivity against K562 of normal spleen cells and TIL**

| Expt No. | % $^{51}Cr$ release mediated by: Spleen cells | TIL | Actual decrease in $^{51}Cr$ release (%)† | Predicted decrease in $^{51}Cr$ release (%)‡ | Differential cell composition of TIL§ TIL | Tumour cells | Monocytes | Polymorphs | Other |
|---|---|---|---|---|---|---|---|---|---|
| 1 | 30 | 14 | 54 | 51 | 90 | 5 | 2 | 2 | 1 |
| 2 | 24 | 20 | 15 | 20 | 94 | 1 | 3 | 2 | 3 |
| 3 | 32 | 26 | 21 | 38 | 94 | 2 | 3 | 0 | 1 |
| 4 | 29 | <1 | 99 | 56 | 88 | 9 | 1 | 1 | 1 |

* Constant E:T ratio, 50:1.

† $\frac{\%\text{ Specific }^{51}\text{Cr release mediated by spleen cells} - \%\text{ specific }^{51}\text{Cr release mediated by TIL}}{\%\text{ specific }^{51}\text{Cr release mediated by spleen cells}} \times 100$

‡ Calculated from Fig. 2.

§ Differential counts were made on Jenner–Giemsa-stained cytocentrifuge films prepared from cell suspension incubated with polystyrene latex for 45 min at 37°C before centrifugation.

FIG. 2.—Effect of viable and formalin-fixed unlabelled Mc40A cells on natural killer (NK) activity of normal rat spleen against K562 targets. Viable Mc40A cells, ○——○ (Mean of 4 experiments ± s.e.); formalin-fixed Mc40A cells, ×——×, ○ - - - ○ and × - - - × (3 separate experiments).

These data indicate that inhibition is not a passive phenomenon caused simply by steric hindrance.

### DISCUSSION

In the assessment of anti-tumour lymphocyte reactivity *in situ*, the recovery of viable and functionally active lymphocytes essentially free from other cellular contaminants is obligatory. In this study Sephadex G-10 column fractionation was employed, using the method originally described by Ly & Mishell (1974) for separation of antibody-forming cells from immune spleen-cell populations. The technique was modified in that nylon wool was used to support the Sephadex instead of glass beads. The original method has been demonstrated to remove adherent phagocytic cells (Pollack *et al.*, 1976) and tumour cells (Hansen *et al.*, 1977), and to yield an almost exclusively lymphocytic eluate retaining the functional attributes of T lymphocytes, K and NK cells (Wolfe *et al.*, 1977). The purity of the eluates originating from the disaggregated Mc40A suspensions was essentially similar to the MSV system (Gillespie *et al.*, 1977) provided that 2 columns were used in sequence. Even with this proviso, a small degree of tumour-cell contamination was unavoidable. However, probably the greatest limitation was the recovery which amounted to only 30% of the pre-fractionation population. The adventitious removal of cells of potential importance in cytotoxic reactions is thus a possibility which must be considered in the interpretation of our data.

The use of different assays for the analysis of tumour-related and non-specific effector functions was necessitated by several considerations. Preliminary efforts to establish a short-term $^{51}Cr$-release assay using Mc40A targets were effectively thwarted by the unpredictable extent of spontaneous isotopic release (28–60% over 18 h), an experience not encountered with the K562 targets used to monitor NK function. Moreover, although cytolysis could be detected in normal spleen-cell populations (Potter & Moore, 1978), increased cytotoxicity in the lymphoid tissues of tumour-bearers compared with those from normal controls could not be unequivocally established. In these circumstances tumour-related activity was sought by the $^{75}Se$ assay.

This assay was adopted because of the greater sensitivity of longer-term assays, which detect both cytolytic and cytostatic effects. An important finding was that after 48 h it was impossible to identify the tumour cells which had initially contaminated the TIL preparations. This could be interpreted in 2 ways: either effete tumour cells, damaged by the isolation procedure, died early in the assay and did not interfere with the cytotoxic activity of lymphocytes in the long term, or they were initially present in a viable state and were subsequently killed by the presence of effector cells in the TIL preparation (Berczi *et al.*, 1973). Under the latter circumstances tumour cells might have competitively inhibited the cytolysis of target cells, as has been demonstrated in short-term assays (Gillespie *et al.*, 1977).

However, if competitive inhibition had been significant in the longer-term $^{75}$Se assay, the effect must have been fortuitous, because each time a TIL population was tested the level of cytotoxicity was comparable with that of normal splenic lymphocytes, regardless of the degree of tumour-cell contamination and of the tumour of origin of the TIL. Thus, by contrast with the $^{51}$Cr assay against K562 targets (discussed below), it is improbable that competitive inhibition is a major complicating factor in the interpretation of our $^{75}$Se data.

Use of the $^{75}$Se assay demonstrated the importance of investigating the relative susceptibility of target cell lines to the cytotoxic effect of lymphoid cells. Thus the results from testing effector cells isolated from the tumour and lymphoid tissue of Mc40A tumour-bearing animals against a range of antigenically distinct tumour target cells could be interpreted as indicating a degree of tumour-specific cytotoxicity. However, the reality of the situation was only revealed when (a) effector cells from normal lymphoid tissue were tested and (b) crossover experiments were performed using TIL isolated from an antigenically distinct sarcoma. These data indicated that target-cell susceptibility, rather than antigenic specificity, influenced the outcome of the cytotoxicity assays.

The anatomical distribution of cytotoxic effector cells and the differential susceptibility of target cells detected by the $^{75}$Se assay suggested that the effector cell in populations derived from Mc40A tumour-bearers was comprised, in part if not wholly, of NK cells. This was confirmed by the lytic activity of the preparations against the xenogeneic K562 target cells. However, an adherent cell also appeared to be active in the $^{75}$Se assay, since Sephadex G-10 filtration reduced the cytotoxicity of both normal and tumour-bearer spleen-cell populations. This cell, putatively a macrophage, may have been similar to the phagocytic, non-specifically cytostatic cell type identified in the spleens of tumour-bearing mice (Mantovani *et al.*, 1977). In that study the presence of tumour was a prerequisite for demonstration of this activity, in contrast to the present findings, where it was also detected in normal spleen. Such a cell, being cytostatic as opposed to cytolytic, would not be expected to be involved in a $^{51}$Cr-release assay, and this was shown to be the case because Sephadex G-10 filtration of spleen cells enhanced $^{51}$Cr release from K562 cells, presumably by removal of irrelevant cells from the effector population.

While Sephadex G-10 treatment reduced the cytotoxicity of both normal and tumour-bearer spleens in the $^{75}$Se assay, the latter remained persistently more cytotoxic than the former. This suggests that a third cell type may be involved in mediating the enhanced cytotoxicity of tumour-bearer spleen cells between Days 15 and 28 of tumour growth. In so far as Sephadex G-10 eluates consist predominantly of T-lymphocytes, and comparable cytotoxicity could not be demonstrated against an antigenically unrelated target (Mc57), the data are consistent with a tumour-related (? specific) component in addition to the NK element. However, this interpretation must be considered with the awareness that Mc57 targets are less susceptible than Mc40A to NK cells.

The close correspondence between the activity of TIL and of normal spleen cells, and their lack of target-cell specificity in the $^{75}$Se assay, implied that the *predominant* effector function in both populations is mediated by NK cells. The finding that the activity of TIL against K562 was lower than that of tumour-bearer spleen cells was thus somewhat unexpected, particularly as the Sephadex G-10 procedure produced a modest but significant *increase* in activity. An alternative possibility that the low levels of cytotoxicity mediated by TIL might be a consequence of nonspecific activation during the separation procedure of an otherwise non-cytotoxic population seems unlikely, but cannot be easily dismissed (Wardley *et al.*, 1976). To do so would require the use of other techniques for the separation of TIL (*e.g.* density-gradient separation) which to date have proved unsatisfactory.

The essence of the phenomenon was disclosed by the inverse relationship between tumour cell contaminants in the TIL population and the level of cytotoxicity relative to that of normal spleen cells. This was confirmed by experiments in which the presence of third-party Mc40A cells caused a dose-dependent decrease in NK activity. It has been shown previously (Potter & Moore, 1978) that Mc40A cells are susceptible to NK effects, so that the phenomenon here described is one of cold inhibition, an interpretation supported by the failure of fixed Mc40A cells to achieve the same effect. In our experience, interference by contaminating tumour cells was thus a more pervasive problem in the short-term cytolytic test than in the longer-term $^{75}$Se assay. Our data indicate that if it had been possible to prepare TIL in which tumour-cell contamination was $<2\%$, their activity would have been at least equal to that of either normal or tumour-bearer spleen cells, estimated by $^{51}$Cr release.

In the rat, as in other species, NK cells are characterized by operational criteria. Although association with a minority population within the TIL compartment cannot be excluded, it is likely that the effectors belong to the predominant population which stains with ATS. Antisera prepared by conventional procedures, like our reagent, have been shown to react with NK cells (Shellam, 1977). Such reactivity is removed by an additional absorption step using spleen cells from thymectomized, irradiated and marrow-reconstituted rats, which leaves anti-T-cell activity intact. Whether the presence of NK cells within tumours has an immunological basis cannot be determined, since little is known at present of the relationship between NK function and conventional immune responses.

It has been postulated that NK cells may play a role in the immune surveillance of tumours (Herberman & Holden, 1978). Recent observations that NK activity is strongly augmented *in vivo* by injection of agents with potent anti-tumour properties such as BCG, *C. parvum* and poly-IC are consistent with this hypothesis (Tracey *et al.*, 1977; Wolfe *et al.*, 1977; Oehler *et al.*, 1978*b*).

However, macrophages also comprise a significant proportion of the infiltrating cells which can be separated from developing tumours (Moore & Moore, 1977*b*); moreover, these cells are functionally active and inhibit the growth of tumour cells as assessed by the $^{75}$Se assay (Moore & Moore, 1979). A role for the macrophage in the regulation of NK activity at a systemic level has been suggested (Oehler & Herberman, 1978) but whether any relationship exists at the tumour site is unknown. The appearance of various host-cell populations in tumours strongly suggests that the actual events *in vivo* are dependent upon precisely controlled relationships between the infiltrating cell types. Probably in no situation is this more apparent than the MSV tumour–host system, where the cytolytic activity of both intra-tumour T lymphocytes (Holden *et al.*, 1976; Gillespie *et al.*, 1977; Plata & Sordat, 1977) and macrophages (Russell *et al.*, 1977; Russell & McIntosh, 1977) varies with the growth status of neoplasms. For instance, specifically cytotoxic T cells can be isolated from both regressors and progressors, but in the latter only during the early stages of tumour growth (Gillespie *et al.*, 1977). Although cytotoxicity data were unattainable before Day 11 of the growth of Mc40A, this system is analogous to that of MSV, in so far as no specifically cytotoxic T cells could be detected in TIL isolated from progressive tumours. Yet there is little doubt that thymus-derived lymphocytes are of primary importance in tumour recognition and rejection, as indicated by the specificity of *in vivo* rejection of small inocula in pre-immunized hosts. However, the vast majority of T lymphocytes presumably enter an established tumour indiscriminately, while only a small proportion may be sensitized to the eliciting antigen and accumulate preferentially. Also their cytotoxic efficiency may be suppressed by factors at a local and/or systemic level.

This work was supported by grants from the Medical Research Council and the Cancer Research Campaign.

### REFERENCES

ALEXANDER, P., ECCLES, S. A. & GAUCI, C. L. L. (1976) The significance of macrophages in human and experimental tumours. *Ann. N.Y. Acad. Sci.*, **276**, 124.

BERCZI, I., STRAUSBAUCH, P. & SEHON, A. H. (1973) Rejection of tumour cells *in vitro*. *Science*, **180**, 1289.

BIDDISON, W. E., PALMER, J. C., ALEXANDER, M. E., COWAN, E. P. & MANSON, L. A. (1977) Characterisation and specificity of murine anti-tumour cytotoxic effector cells within an ascitic tumour. *J. Immunol.*, **118**, 2243.

BROOKS, C. G., REES, R. C. & ROBINS, R. A. (1978) Studies on the microcytotoxicity test. II. The uptake of amino acids ([$^3$H] leucine or [$^{75}$Se] methionine) but not nucleosides ([$^3$H] thymidine or [$^{125}$I] IUDR) or $^{51}$Cr $O_4{}^{2-}$ provides a direct and quantitative measure of target cell survival in the presence of lymphoid cells. *J. Immunol. Methods*, **21**, 111.

ECCLES, S. & ALEXANDER, P. (1974) Macrophage content of tumours in relation to metastatic spread and host immune reaction. *Nature*, **250**, 667.

EVANS, R. (1972) Macrophages in syngeneic animal tumours. *Transplantation*, **14**, 468.

EVANS, R. (1973*a*) Preparation of pure cultures of tumour macrophages. *J. Natl Cancer Inst.*, **50**, 271.

EVANS, R. (1973*b*) Macrophages and the tumour-bearing host. *Br. J. Cancer*, **28**, Suppl. I, 19.

GILLESPIE, G. Y., HANSEN, C. B., HOSKINS, R. G. & RUSSELL, S. W. (1977) Inflammatory cells in solid murine neoplasms. IV. Cytolytic T lymphocytes isolated from regressing and progressing Moloney sarcomas. *J. Immunol.*, **119**, 564.

HANSEN, C. B., GILLESPIE, G. Y. & RUSSELL, S. W. (1977) Isolation of T-lymphocytes from disaggregated tumours, with high purity and good percentage recovery. *J. Natl Cancer Inst.*, **59**, 273.

HASKILL, J. S., PROCTOR, J. W. & YAMAMURA, Y. (1975*a*) Host responses within solid tumours. I. Monocytic effector cells within rat sarcomas. *J. Natl Cancer Inst.*, **54**, 387.

HASKILL, J. S., YAMAMURA, Y. & RADOV, L. (1975*b*) Host responses within solid tumours: Non-thymus-derived specific cytotoxic cells within a murine mammary adenocarcinoma. *Int. J. Cancer*, **16**, 798.

HERBERMAN, R. B. & HOLDEN, H. T. (1978) Natural cell-mediated immunity. *Adv. Cancer Res.*, **27**, 305.

HOLDEN, H. T., HASKILL, J. S., KIRCHNER, H. & HERBERMAN, R. B. (1976) Two functionally distinct anti-tumour effector cells isolated from primary murine sarcoma virus-induced tumours. *J. Immunol.*, **117**, 440.

KERBEL, R. S., PROSS, H. F. & ELLIOT, E. V. (1975) Origin and partial characteristation of Fc receptor bearing cells found within experimental carcinomas and sarcomas. *Int. J. Cancer*, **15**, 918.

LY, I. A. & MISHELL, R. I. (1974) Separation of mouse spleen cells by passage through columns of Sephadex G-10. *J. Immunol. Methods*, **5**, 239.

MANTOVANI, A., EVANS, R. & ALEXANDER, P. (1977) Non-specific cytotoxicity of spleen cells in mice bearing transplanted chemically-induced fibrosarcomas. *Br. J. Cancer*, **36**, 35.

MOORE, K. & MOORE, M. (1977*a*) Intra-tumour host cells of transplanted rat neoplasms of different immunogenicity. *Int. J. Cancer*, **19**, 803.

MOORE, M. & MOORE, K. (1977*b*) Kinetics of macrophage infiltration of experimental rat neoplasms. In *The Macrophage and Cancer*. Eds K. James, B. McBride & A. Stuart. University of Edinburgh. p. 330.

MOORE, M. & MOORE, K. (1979) *In situ* expressions of anti-tumour immunity. In *Contemporary Topics in Immunobiology*. (In press).

OEHLER, J. R., LINDSAY, L. R., NUNN, M. E. & HERBERMAN, R. B. (1978*a*) Natural cell-mediated cytotoxicity in rats. I. Tissue and strain distribution and demonstration of a membrane receptor for the Fc portion of IgG. *Int. J. Cancer*, **21**, 204.

OEHLER, J. R., LINDSAY, L. R., NUNN, M. E., HOLDEN, H. T. & HERBERMAN, R. B. (1978*b*) Natural cell-mediated cytotoxicity in rats. II. *In vivo* augmentation of NK-cell activity. *Int. J. Cancer*, **21**, 210.

OEHLER, J. R. & HERBERMAN, R. B. (1978) Natural cell-mediated cytotoxicity in rats. III. Effects of immunopharmacologic treatments on natural reactivity and on reactivity augmented by polyinosinicpolycytcylic acid. *Int. J. Cancer*, **21**, 221.

PLATA, F. & SORDAT, B. (1977) Murine sarcoma virus (MSV)-induced tumours in mice. I. Distribution of MSV-immune cytolytic T lymphocytes *in vivo*. *Int. J. Cancer*, **19**, 205.

POLLACK, S. B., NELSON, K. & GRAUSZ, J. D. (1976) Separation of effector cells mediating antibodydependent cellular cytotoxicity (ADC) to erythrocyte targets from those mediating ADC to tumour targets. *J. Immunol.*, **116**, 944.

POTTER, M. R. & MOORE, M. (1978) Organ distribution of natural cytotoxicity in the rat. *Clin. Exp. Immunol.*, **34**, 78.

PROSS, H. F. & KERBEL, R. S. (1976) An assessment of intra tumour phagocytic and surface marker bearing cells in a series of autochthonous and early passaged chemically-induced murine sarcomas. *J. Natl Cancer Inst.*, **57**, 1157.

RUSSELL, S. W., DOE, W. F., HOSKINS, R. G. & COCHRANE, C. G. (1976*a*) Inflammatory cells in solid murine neoplasms. I. Tumour disaggregation and identification of constituent inflammatory cells. *Int. J. Cancer*, **18**, 322.

RUSSELL, S. W., GILLESPIE, G. Y., HANSEN, C. B. & COCHRANE, C. G. (1976*b*) Inflammatory cells in solid murine neoplasms. II. Cell types found throughout the course of Moloney Sarcoma regression or progression. *Int. J. Cancer*, **18**, 331.

RUSSELL, S. W., DOE, W. F. & MCINTOSH, A. T. (1977) Functional characterisation of a stable, non-cytolytic stage of macrophage activation in tumours. *J. Exp. Med.*, **146**, 1511.

RUSSELL, S. W. & MCINTOSH, A. T. (1977) Macrophages isolated from regressing Moloney sarcomas are more cytotoxic than those recovered from progressing sarcomas. *Nature*, **268**, 69.

SHELLAM, G. R. (1977) Gross-virus-induced lymphoma in the rat V. Natural cytotoxic cells are non-T cells. *Int. J. Cancer*, **19**, 225.

TRACEY, D. E., PROSS, H. F., JONDAL, M. & WITZ, I. P. (1975) Antibody-dependent cell-mediated cytotoxic activity in syngeneic mouse ascites tumours. *Int. J. Cancer*, **16**, 870.

TRACEY, D. E., WOLFE, S. A., DURDICK, J. M. & HENNEY, C. S. (1977) BCG-induced murine effector cells. I. Cytolytic activity in peritoneal exudates: An early response to BCG. *J. Immunol.*, **119**, 1145.

VAN LOVEREN, H. & DEN OTTER, W. (1974) Macrophages in solid tumours. I. Immunologically specific effector cells. *J. Natl Cancer Inst.*, **53**, 1057.

WARDLEY, R. C., ROUSE, B. T. & BABIUK, L. A. (1976) Lymphocyte activation by cell separation procedures. *Immunol. Commun.*, **5**, 637.

WOLFE, S. A., TRACEY, D. E. & HENNEY, C. S. (1977) BCG-induced murine effector cells. I.. Characterisation of natural killer cells in peritoneal exudates. *J. Immunol.*, **119**, 1152.

WOOD, G. L. & GILLESPIE, Y. (1975) Studies on the role of macrophages in regulation of tumour growth and metastasis of murine chemically-induced tumours. *Int. J. Cancer*, **16**, 1022.

WOOD, G. L., GILLESPIE, G. Y. & BARTH, R. F. (1975) Receptor sites for antigen–antibody complexes on cells derived from solid tumours: detection by means of antibody-sensitised sheep erythrocytes labelled with Technetium-99m. *J. Immunol.*, **114**, 950.